# Multiplex multi-antigen, multi-species, microsphere-based ELISA to detect antibodies to three human *Plasmodium* species

**DOI:** 10.1186/1475-2875-11-S1-P119

**Published:** 2012-10-15

**Authors:** Babita Mahajan, Victoria F Majam, Prasun Moitra, Yukiko Kozakai, Phuong T Pham, Hong Zheng, David E Lanar, David L Narum, Isabella Quakyi, Sanjai Kumar

**Affiliations:** 1 Laboratory of Emerging Pathogens, Division of Emerging and Transfusion Transmitted Diseases, OBRR, FDA, Rockville, Maryland, 20852, USA; 2 Division of Malaria Vaccine Development, Walter Reed Army Institute of Research, Silver Spring, Maryland, 20910, USA; 3 Laboratory of Malaria Immunology and Vaccinology National Institute of Allergy and Infectious Diseases, National Institutes of Health, Rockville, Maryland, 20892, USA; 4 School of Public health, University of Accra, Accra, Ghana

## Background

Multiplex ELISA that detect antibodies against more than one *Plasmodium* species while allowing species differentiation would be highly valuable for epidemiology and vaccine studies in areas of mixed infections and identification of malaria-exposed blood donors in non-endemic countries. Here we report a highly sensitive, multiplex ELISA based on recombinant proteins from *Plasmodium falciparum*, *P. vivax and P. malariae* malaria for pan-species and species-differentiating detection of antibodies in malaria-positive reference samples and in samples from individuals living in a malaria endemic region in Ghana, Africa.

## Materials and methods

Multiplex ELISA was developed utilizing the Luminex xMAP technology that allows the simultaneous detection of antibodies of different specificities that react with antigenic epitopes on multiple beads (microspheres) of different dye intensity. Seven recombinant Plasmodium antigens (P. *falciparum*: CSP, AMA-1, LSA-1 and MSP1_19_, *P. vivax*: AMA-1 and MSP1_19_ and *P. malariae:* MSP1_19_) were covalently coupled to carboxylated magnetic beads. The dilutions of human plasma/serum were incubated with 3000-5000 antigen-conjugated beads in 96-well plate. Following incubation with a biotin-labeled human anti-IgG conjugate, a streptavidin-PE conjugated fluorescent substrate was added and the plates were read on Bio-Rad BioPlex 200 reader. The reader was set to read a minimum of 50 beads with identical unique detection signal, the results were expressed as median-fluorescent intensity and cut-off titers were established using a pool of normal human serum samples from the US blood donors.

## Results

Multiplex ELISA detected 100% of the confirmed malaria reference samples belonging to *P. falciparum*, *P. vivax* and *P. malariae* infected patients. The inclusion of multiple antigens in the multiplex assay makes the test more sensitive than the conventional plate ELISA. The assay was capable to detect differential antibody reactivity to seven *Plasmodium* antigens in serum samples from 75 adults from malaria endemic area in Ghana who had no demonstrable parasitemia by microscopy. The assay also successfully distinguished between the mixed *P. falciparum* and *P. malariae* infections in imported malaria samples obtained in United States.

## Conclusions

We have developed a highly sensitive multiplex ELISA that detects the antibodies specific to *P. falciparum*, *P. vivax* and *P. malariae* in a single test format. This assay is being further improved to incorporate the fourth human *Plasmodium* - *P. ovale.* We think that this test may be of high value in epidemiological surveys to determine species-specific malaria exposure in areas of mixed infections and vaccine efficacy studies.

